# Microbial Typing by Machine Learned DNA Melt Signatures

**DOI:** 10.1038/srep42097

**Published:** 2017-02-06

**Authors:** Nadya Andini, Bo Wang, Pornpat Athamanolap, Justin Hardick, Billie J. Masek, Simone Thair, Anne Hu, Gideon Avornu, Stephen Peterson, Steven Cogill, Richard E. Rothman, Karen C. Carroll, Charlotte A. Gaydos, Jeff Tza-Huei Wang, Serafim Batzoglou, Samuel Yang

**Affiliations:** 1 Emergency Medicine, Stanford University, Stanford, California, 94305, USA; 2 Computer Science, Stanford University, Stanford, California, 94305, USA; 3 Biomedical Engineering, The Johns Hopkins University, Baltimore, Maryland, 21218, USA; 4 Infectious Disease, Medicine, The Johns Hopkins University, Baltimore, Maryland, 21218, USA; 5 Emergency Medicine, The Johns Hopkins University, Baltimore, Maryland, 21218, USA; 6 Medical Microbiology, Pathology, The Johns Hopkins University, Baltimore, Maryland, 21218, USA; 7 Mechanical Engineering, The Johns Hopkins University, Baltimore, Maryland, 21218, USA

## Abstract

There is still an ongoing demand for a simple broad-spectrum molecular diagnostic assay for pathogenic bacteria. For this purpose, we developed a single-plex High Resolution Melt (HRM) assay that generates complex melt curves for bacterial identification. Using internal transcribed spacer (ITS) region as the phylogenetic marker for HRM, we observed complex melt curve signatures as compared to 16S rDNA amplicons with enhanced interspecies discrimination. We also developed a novel Naïve Bayes curve classification algorithm with statistical interpretation and achieved 95% accuracy in differentiating 89 bacterial species in our library using leave-one-out cross-validation. Pilot clinical validation of our method correctly identified the etiologic organisms at the species-level in 59 culture-positive mono-bacterial blood culture samples with 90% accuracy. Our findings suggest that broad bacterial sequences may be simply, reliably and automatically profiled by ITS HRM assay for clinical adoption.

Acute febrile illnesses often present with vague symptoms and are caused by a myriad of potential bacterial pathogens^1^,^2^,^3^. Unfortunately, accurate diagnosis of the causative agent(s) can be compromised by diagnostic assays biased to target a narrow pre-determined set of microorganisms. Broad differential diagnostics that rapidly identify unknown bacterial pathogen(s) within the acute-care timescale would enable early fine-tuning of antibiotic stewardship and reduce patient morbidity and mortality^4^.

Molecular approaches based on broad-range nucleic acid amplification of bacterial targets are well suited for differential diagnosis of acute febrile illness. However, downstream amplicon analysis technologies (e.g. microarray, mass spectrometry, and sequencing) for microbial identification vary in information content, complexity, speed and cost, factors which limit their practicality for clinical implementation. High Resolution Melt (HRM) interrogates an amplicon’s sequence variants through heat denaturation in the presence of an intercalating dye. This technique takes place as a rapid single-step, closed-tube process performed directly on generic PCR platforms^5^. Although not as information rich as sequencing, the simplicity, speed, cost, and accessibility of HRM suggest that microbiological analysis protocols that incorporate HRM for first-pass screening or diagnosis hold great promise for clinical adoption. Due to these advantages, our goal is to develop HRM-based strategies to accomplish reliable sequence fingerprinting for bacterial species identification.

In HRM, the pattern of an amplicon’s heat denaturation generates a sigmoidal melt curve. The peak of the curve’s first derivative dictates the melting temperature (Tm), which is primarily determined by the sequence %GC content and length. Previous HRM studies used short amplicons (<300 bp) and found that small (0.2 °C) shifts in Tm between samples generated reliable variant discrimination^6^. However, short amplicons tend to generate Tms with narrow temperature ranges (~4 °C based on our prior work^7^) of the full melt spectrum (i.e. 60–95 °C). If 0.2 °C is required to identify differences, a dynamic Tm range of 4 °C could only distinguish up to 20 variants. This represents a major challenge if HRM is to be expanded for large-scale fingerprinting of potentially hundreds of sequences. To overcome this limitation, we and others have designed HRM assays using multiple separate amplification reactions and primer sets to interrogate short hypervariable sequence stretches within the 16S rRNA gene (16S)^7^,^8^,^9^,^10^,^11^,^12^,^13^,^14^. This multiplex approach may moderately improve the discriminatory power, but the multiple parallel reactions complicate assay format and are impractical for samples containing few targets.

In addition to Tm-based information, HRM also observes the exact melt curve shape as a function of the actual nucleic acid sequence and strand complementarity. Multiphasic melt curves can arise depending on the number of regions with different %GC content (melt domains), which is beneficial in sequence fingerprinting^15^. We explored the use of a long amplicon (~1000 bp) covering six hypervariable regions in the 16S genetic locus to gather more melt domains and sequence diversity with the use of a single primer set^16^. Although the long amplicons yielded more biphasic melt curves, with occasional double “peaks” on the derivative curves, the narrow Tm range still constrains the profiling breadth. The 16S also has limited sequence variability to enable species-level discrimination of organisms (only 65 to 83% of cases)^17^. Another contributor to curve shape is the heteroduplex that forms when two strands from different amplicons with sufficient base complementarity anneal during cooling. Artificial heteroduplex formation by mixing reference DNA with unknown DNA in a 1:1 ratio has been exploited as a strategy for heterozygote and variant screening to enhance melt curve diversity^18^. However, this multi-step approach adds to assay complexity. Herein, we explore the use of bacterial internal transcribed spacer region (ITS) as an alternative target locus. We hypothesize that by strategically choosing this phylogenetic locus for both its higher interspecies and intragenomic sequence heterogeneity, we can generate highly complex melt curves for large-scale profiling with enhanced species-level specificity in a simple assay format (Fig. 1).

HRM sensitivity to subtle differences in experimental conditions such as inconsistencies in instrumental operation and pipetting often causes run-to-run melt curves variability. The current HRM data analysis, performed with the accompanying instrument software or a commercially available one such as ScreenClust^19^, is not capable of compensating for these fluctuations and therefore diminish the HRM assay’s discriminatory power. To address this issue, we have developed an adaptive Naïve Bayes algorithm which has the capability to 1) enable automated melt curve classification with trained tolerance for variations in experimental conditions, 2) use a database of melt curves from reference bacterial organisms for subsequent curve-matching analysis of unknown samples, 3) discover unanticipated organisms when no match is found in the melt curve database, and 4) provide statistical interpretation. These features will yield clinically relevant information through unbiased automated interrogation of our HRM assay results.

## Results

### Targeting ITS generates high complexity melt curves

The 16S–23S ribosomal DNA (rDNA) internal transcribed spacer region (ITS) of the ribosomal operon *rrn* was used as an alternative to traditional 16S rDNA ribotyping for phylogenetic classification. ITS is known to have substantially greater interspecies polymorphisms yet high intraspecies conservation^20^. It is approximately 60–1,500 bp in length with up to 15 copies per genome and intragenomic variations in terms of length and sequence. Since ITS features may affect melt curve conditions, we hypothesized that ITS is an ideal target for HRM analysis that will generate melt curves of complex shapes.

**Figure 2:**
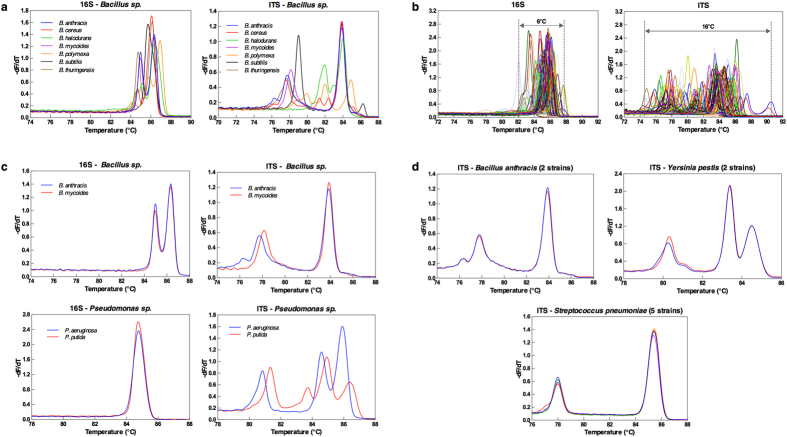
ITS Provides High Information Profiles Inclusive to the Species Level. (**a**) Representative 16S and ITS melt curves of seven (7) species of the Bacillus genus. (**b**) 16S and ITS composite melt curves of all 89 bacterial organisms in our library. (**c**) ITS melt curves from a single strain of *B. anthracis, B. mycoides, Pseudomonas aeruginosa, P. putida* are compared to the their undistinguishable 16S counterparts. (**d**) ITS melt curves from two (2) strains of *B. anthracis*, 2 strains of *Y. pestis*, and five (5) strains of *S. pneumoniae* are used for the strain inclusivity test.

To evaluate this, we generated a library of both long 16S and ITS amplicons for 89 available and characterized bacterial species archived from our prior studies. The library included mostly one strain per species with the exception of several species as listed and noted in Supplementary Table S1. These 89 species covered common pathogenic and commensal bacteria across multiple phylogenetic families and genera. Representative derivative curves for each species are available in Supplementary Fig. S1. All species exhibit high diversity in curve shapes within a given genus (i.e. *Bacillus*) (Fig. 2a) as well as across genera (Fig. 2b). Increased diversity of ITS curve shape as compared to 16S is clearly evident, with the ITS curves showing multiple peaks, and a much wider Tm range (15 °C compared to 5 °C for 16S) (Fig. 2a–d). We also observed that in our library, some species of the same genus that were indistinguishable by their 16S curves before can now be differentiated by their ITS curves (Fig. 2c). Multiple strains for several species included in our library generated identical melt curves, suggesting strain inclusivity at the species-level for identification (Fig. 2d). We also confirmed run-to-run and inter-operator reproducibility.

### Template concentration on ITS curve integrity

To assess the impact of template concentration on ITS curve integrity, we serially diluted *E. coli* genomic DNA based on it genome copies down to extinction and performed either 40-cycle or 50-cycle PCR targeting the ITS region. At template concentrations below 10^2^ genome copies per reaction, peak height of melt curves decreased in 40-cycle PCR HRM but was retained down to 1 genome copy in 50-cycle PCR HRM (Supplementary Fig. S2a and b).

### Heteroduplex analysis

**Figure 3:**
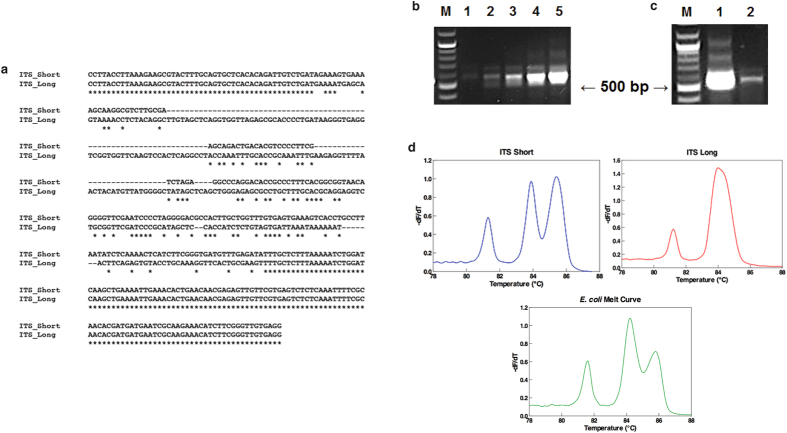
Heteroduplex analysis of *E. coli* ITS. (**a**) Clustal Omega Multiple Sequence Alignment of *E. coli* ATCC 25922 (GenBank Accession number CP009072) ITS short (361 bp) and ITS long (453 bp) sequences. (**b**) ITS homoduplex-heteroduplex profiles obtained after 20 (1), 25 (2), 30 (3), 35 (4) and 40 (5) number of PCR cycles. Slow migrating bands (at 800 and 1000 bp) were visible starting from cycle number 25, suggesting the heteroduplex nature of the bands. Expected homoduplex bands were at 540 bp (361 bp ITS short + 179 bp of 3′ of 16S and 38 bp of 5′ 23S), and 632 bp (453 bp + 179 bp). Lane M contains 100-bp DNA marker. (**c**) Agarose gel electrophoresis showing *E. coli* ITS PCR products treated (2) and untreated (1) with mung bean nuclease, an enzyme that recognizes and cleaves single stranded DNA, even when it is located in double-stranded DNA products. The loss of the higher molecular weight bands confirms the heteroduplex nature of the bands, leaving the true homoduplexes. Lane M contains 100-bp DNA marker. (**d**) ITS HRM analysis on 20 colonies resulted in 2 distinct melt curve groups (ITS short and ITS long). Combinations of the amplicons from each group did not recreate the original *E. coli* melt curve.

In order to determine if heteroduplex amplicon formation due to intragenomic sequence variants is the primary contributing factor to complex ITS melt curves observed, we first examined the entire genome of a representative *E. coli* strain (strain ATCC 25922, GenBank Accession number CP009072), and found 7 ITS copies characterized by 6 copies containing the same shorter sequence (ITS short) and 1 copy containing a longer sequence (ITS long). Alignment of these two sequences using Clustal Omega^21^ *in silico* revealed multiple regions of heterologous internal sequence (Fig. 3a). We then amplified the ITS region of *E. coli* ATCC 25922 with increasing cycle number. A slower migrating band observed on the gel electrophoresis beyond 25 cycles suggested increasing presence of heteroduplex structures (Fig. 3b). This was confirmed through subsequent treatment of ITS amplicons with mung bean nuclease, which cleaves unpaired DNA strands of heteroduplex products, resulting in loss of the larger band (Fig. 3c). We also performed ITS HRM analysis on 20 *E. coli* ITS clones from which we observed two distinct melt curve profiles (Fig. 3d). Sequencing of the ITS inserts corresponding to these two different melt curve profiles matched to either ITS short or ITS long sequence in a 3:1 distribution ratio respectively. Melt profile of the more dominant shorter ITS clone alone revealed the 3 melt peaks similar to the original *E. coli* ITS melt curve. Only 2 melt peaks were observed in the longer ITS clones. Mixing amplicons from each group in various DNA concentration ratios followed by HRM assay did not contribute to additional melt peaks.

### Development of classification algorithm

Our ITS melt curve library was generated from the 89 aforementioned bacterial species and an additional 10 randomly selected clinical positive blood culture samples that represented 10 different species that we had multiple samples of. Using this library, we performed a supervised classification analysis by training the proposed adaptive Naïve Bayes algorithm on a reference panel and testing the classifier on a separate panel of unknown samples. The goal is two-fold: 1) to predict whether the curve for the unknown sample belongs to those in the reference panel; if so, 2) to determine the species identity of the unknown sample. Our algorithm adaptively introduces an auxiliary label indicating the possibility of the tested sample to be unrepresented in the reference panel. This mechanism enables us to reliably identify novel samples, which have not been identified before. Our algorithm first calculates curve similarities based on both curve shapes and curve peak positions. Hilbert transform is used to enhance the similarity. With reliable similarity metric, our algorithm seeks nearest samples in the reference panel and constructs a probabilistic decision boundary based on the similarities to the test sample. Leave-one-out cross validation (LOOCV) analysis was performed on the reference panel with this algorithm by testing one replicate of a strain against the rest of the panel including the other replicates. This analysis revealed that 95% of species curves were reliably identified. Reference data and training results are available upon request.

### ITS HRM assay on positive blood culture samples

To measure the accuracy of our ITS HRM assay (ITS PCR HRM analysis coupled with classification algorithm), we tested our assay against a convenience sample of 87 de-identified residual positive-blood-culture specimens prospectively collected from the clinical microbiology laboratory. Ten of the 87 samples were used for training our algorithm as mentioned in the previous section. Seventeen of the remaining 77 samples were omitted due to poly-bacterial or yeast culture results. We then performed 16S sequencing on 60 samples. The culture and sequencing results matched for 59 out of 60 samples. We omitted the sample with the discordant culture and 16S sequencing results, leaving us with 59 test samples representing 21 different bacterial species of which 5 species were not in our database.

We performed the ITS HRM assay on these 59 samples in duplicate, and utilized the 16S sequencing results as the reference for correct melt curve classification. Our classification algorithm incorporated both duplicate melt curves of a sample for analysis and determined the top 2 curve matches accompanied by their respective posterior probability values. Considering only the top 1 match, the algorithm correctly classified 53 samples and misclassified 6 samples with 90% classification accuracy. When we include the top 2 matches, the algorithm correctly classified 54 samples resulting in 92% classification accuracy (Table 1).

## Discussion

Our single-plex ITS HRM assay can be easily implemented to identify unknown bacterial species by observing their highly variable melt curve shapes with multiple peaks and wide Tm range (15 °C). In this study, we successfully differentiated and generated a training library of unique melt profiles for 89 different clinically relevant bacterial species, some from closely related species of the same genera. Based on this library, our ITS HRM assay correctly identified 59 positive blood culture bottles with 6 discrepancies for a total combined accuracy of 95% at the genus-level and 90% at the species-level. This included correct classification of 5 unanticipated species not included in our library. Of note, our algorithm identified two of the discrepant samples as *Staphylococcus epidermis and Staphylococcus caprae* whereas 16S sequencing identified them as *Staphylococcus hominis.* All three species fall in the coagulase-negative Staphylococcus group, among which the clinical need to identify them to species level remains controversial^22^. Two other discrepant samples were misclassified as unknowns, which in a clinical setting would have required additional analysis such as sequencing or await culture for identification. The overall classification accuracy could likely be improved with further expansion of our training samples.

The ITS sequence between the 16S-23S rRNA is less evolutionarily constrained than its flanking genes^23^, making it better suited as a single species-level phylogenetic locus to simplify assay format for routine clinical adoption. Although our library of available reference species is not exhaustively comprehensive but still reasonably broad, our preliminary results suggest greater resolving power of ITS over 16S HRM analysis in species-level discrimination for multiple genera. Heteroduplex formation of ITS amplicons due to intragenomic sequence variants was demonstrated; however, contrary to our hypothesis, it is unlikely the main contributor to curve shape complexity. We postulate that multiple melt domains harbored within the short (361 bp) ITS sequences are more likely the major contributing factor to multiple melt peaks observed^5^. Future work is needed to assign melt domains to sequence domains. One potential advantage of whole amplicon HRM for microbial typing over probe-based or other sequence fingerprinting approaches capable of resolving single nucleotide polymorphisms is its tolerance for small intraspecies sequence differences^24^. We did observe identical intraspecies melt profiles based on very limited number of strains for several species in our test panel. However, previous studies had targeted ITS for strain differentiation within one bacterial species^25^,^26^. Although curve difference is more prominent between species, ITS was shown to have enough intraspecies sequence and copy number variation to produce strain specific melt curves^27^. This may limit our assay capability to identify a certain type of strain to its correct species if the strain variation is not in our database. Further testing with expanded number of species and their many strains is required to better assess inclusivity. Resulting data sets will also train the algorithm by incorporating subtle strain variation for a robust species-level identification.

Varying template concentrations did not significantly affect overall curve integrity as long as amplicon accumulation reaches plateau phase. Despite improvement in detection limit of our ITS PCR by increasing PCR cycle number, we chose the standard 40-cycle PCR format given considerations for reduced assay time. Subtle variations in peak height due to concentration differences can still be learned and correctly classified by the algorithm.

Our current platform is more reliable for diagnosing mono-bacterial infection. In the case of a poly-bacterial sample, the contribution of individual bacterial populations to the ensemble melt curve is difficult to decouple and identify. We previously developed Universal digital High Resolution Melt (U-dHRM) to overcome this challenge by diluting the starting material such that each genotype is distributed into its own digital PCR reaction and generates a pure melt curve^7^. Targeting the 16S, this approach successfully identified bacterial species at a single molecule level. The ability to quantitatively resolve sample heterogeneity may aid discrimination between true infection versus colonization or contamination, but U-dHRM’s reliance on melt curve Tm limits extent of analysis. For future assessment, replacing 16S rDNA with ITS as the target sequence may considerably increase U-dHRM’s specificity to resolve all species in a mixed sample. The simplicity of our single-plex assay also facilitates scaling to the single cell/molecule format with advanced microfluidic digital droplet platforms^28^, making it highly sensitive to low level targets.

We have developed a new adaptive Naïve Bayes algorithm, which we demonstrate to be capable of differentiating between closely related species, a task previously challenging with 16S rRNA-based methods. Our algorithm consists of three steps, as described in Supplementary Methods. First, we align each curve according to a preset temperature to ensure improved accuracy in the subsequent curve similarity calculation. Second, our algorithm employs a Hilbert transformation of the melt curves, and constructs an enhanced similarity metric between curves. The resulting similarity metric is robust to noise in the curve measurements and contributes to the accuracy of the classifier. Finally, our algorithm performs Naïve Bayes-based classification and reports a final classification results by integrating posterior distributions for all melt curves from one species. Our algorithm is also extended with the function of identifying unseen samples outside the reference panel and reporting them as such. It is important to note that the classification capability of the proposed classifier depends heavily on the amount of training data, and should improve as more data become available^29^.

Overall, our results suggest that ITS HRM assay is a simple, rapid, and practical molecular approach for broad-scale microbial species identification. It is the first to readily identify 89 bacterial species using a single-plex approach, without the need for microbial culture, hybridization probes, mass spectrometry, or sequencing. Our expandable reference database and trainable curve-matching algorithm with statistical interpretation enable discovery of unanticipated organisms with which our database can be updated after sequence verification. Similar use of ITS region may be extended to phylogenetic identification of other organism classes^30^,^31^. We recognize that a larger scale clinical study with a more comprehensive training library of species and strains would be needed to validate our preliminary findings. Nonetheless, as a simple add-on to most commonplace qPCR with HRM capabilities or emerging molecular point-of-care platforms designed for direct sample testing, our ITS HRM assay holds promise for clinical adoption as an early adjunctive test to traditional microbiological methods for etiologic diagnosis of acute febrile illnesses.

## Methods

### Bacterial Genomic DNA for Library Generation

Archived from previous studies, eighty-nine bacterial species were clinically isolated from the clinical microbiology laboratory at Johns Hopkins Hospital or purchased from American Type Culture Collection (ATCC). Bacterial genomic DNA from these species were extracted as previously described^13^.

### PCR HRM Analysis

Real-time PCR amplification and HRM analysis of ITS and 16S rRNA gene was performed on the Rotor-Gene Q thermal cycler (Qiagen, Venlo, Netherlands) with ITS primers pairs ITS1F (5′-TTGTACACACCGCCCG-3′) and ITS2R (5′-YGCCAAGGCATCCACC-3′) and 16S primers pairs V1F (5′-GYGGCGNACGGGTGAGTAA-3′) and V6R (5′-AGCTGACGACANCCATGCA-3′). All standard measures to prevent sample contamination were taken including the use of a designated PCR workstation. PCR reactions were performed in a 20 μL volume using Type-It HRM kit (Qiagen, Venlo, Netherlands) with the addition of 200 nM low temperature calibrator^32^ and 2 μL genomic DNA. Before adding the low temperature calibrator and genomic DNA, each reaction was treated with 0.5 μL of dsDNase and 0.5 μL DTT (ArcticZymes PCR Decontamination Kit, ArcticZymes, Tromsø, Norway) followed by incubation at 37 °C for 20 minutes and 60 °C for 20 minutes to eliminate possible contaminating DNA. Reactions were covered with a 20 μL overlay of PCR grade mineral oil (Fisher Scientific, Hanover Park, IL) and cycling conditions were 95 °C for 5 min, followed by 40 amplification cycles of 95 °C for 15 s, 55 °C for 30 s, and 72 °C for 60 s for ITS or 90 s for 16S, 95 °C for 30 s and 28 °C for 30 s. HRM acquisition at every 0.1 °C immediately followed from 50 °C to 95 °C. The entire run was completed within 3 hours. HRM analysis was performed utilizing the RotorGene-Q software. Raw melt data files were exported and sent for further analysis utilizing the classification algorithm.

### Heteroduplex Analysis

One μg of *E. coli* ATCC 25922 ITS PCR product was added to 25 μL final reaction volume containing 2.5 μL 10x reaction buffer and 1 μL of 10 U/μL mung bean nuclease. The reaction was incubated at 30 °C for 45 minutes. 25 μL of 0.02% SDS was added to stop the nuclease activity. The product was purified using MinElute Reaction Clean Up kit (Qiagen, Venlo, Netherlands) and eluted in 10 μL elution buffer. The purified nuclease treated samples were run in a 1% agarose gel, and imaged. For HRM analysis, ITS PCR reactions were performed as mentioned above and taken out after cycle number 20, 25, 30, 35, and 40. We put the reactions back into the thermal cycler and amplicons were melted from 65 °C–95 °C. Melt profiles were evaluated utilizing the RotorGene-Q software.

For cloning of *E. coli* ITS, the ITS PCR product was purified using MinElute Reaction Clean Up kit (Qiagen, Venlo, Netherlands) and cloned using NEB^®^ PCR Cloning Kit (New England Biolabs, Ipswich, MA) following manufacturer’s protocol. Twenty colonies were picked, screened for insert, and purified for plasmids. Each plasmid was subjected to ITS HRM analysis and the ITS PCR product of representative plasmids of ITS short and ITS long were sent for Sanger sequencing.

### Blood Culture Sample Collection and Preparation

Eighty-seven waste positive blood culture bottles were consecutively collected between July and August 2015 in the clinical microbiology laboratory at Johns Hopkins Hospital according to a protocol approved by the Institutional Review Board of The Johns Hopkins University (IRB NA_00013251). Briefly, blood was drawn from patients as part of routine clinical care for suspected bloodstream infections as recommended by the central hospital laboratory. The clinical blood culture bottles (Bactec Lytic/10 anaerobic medium, the Bactec plus aerobic/F media and the Bactec Standard/10 aerobic medium) were processed using the Bactec FX blood culture continuous monitoring system (bioMérieux, Inc., Durham, NC). None of the bottles tested contained charcoal. Further identification of bacterial isolates was performed using the Phoenix microbial identification system (Becton, Dickinson, Sparks, MD) or Matrix Assisted Laser Desorption Ionization Time of Flight Mass Spectrometry using the Bruker MicroFlex instrument (Bruker Daltonics, Lowell, MA). After reference testing was complete, the remaining waste sample was de-identified for research purposes and stored at 4 **°**C. Pathogens identified by standard microbiological testing were recorded. Explicit consent was not sought, since residual waste samples were retrospectively tested in a de-identified manner. Results from the PCR/HRM test was compared to reference testing results but not used to inform clinical treatment. Bacterial DNA was extracted from 500 μL aliquots of blood culture sample using a previously described protocol based on the Roche MagNA Pure extraction instrument (Roche Diagnostics)^33^. All blood culture samples were initially stored at 4 °C. Processing occurred as quickly as possible, with a maximum of 1 week of storage prior to batch DNA extraction.

### 16S Sequence Analysis

All blood culture samples were amplified for 16S using primers pairs V1F and V6R. The PCR product was purified for sequencing utilizing the Qiagen QIAquick PCR purification kit (Qiagen, Venlo, Netherlands) and sent for Sanger sequencing at MCLAB (San Francisco, CA). The sequencing results (typically 600–700 bp long) were used to choose the best matching database sequence in the NCBI database of 16S sequences using nucleotide BLAST.

### Supervised Validation for Library Generation by Adaptive Naïve Bayes

We have utilized a Naïve Bayes algorithm in order to classify 89 species. The proposed adaptive Naïve Bayes (See Supplementary Methods) is able to predict whether the unknown sample is among the reference panel and subsequently distinguish the species of the sample. We incorporated a low temperature calibrator DNA duplex to correct for tube-to-tube or run-to-run experimental variations for curve alignment and normalization.

## Additional Information

**How to cite this article**: Andini, N. *et al*. Microbial Typing by Machine Learned DNA Melt Signatures. *Sci. Rep.* **7**, 42097; doi: 10.1038/srep42097 (2017).

**Publisher's note:** Springer Nature remains neutral with regard to jurisdictional claims in published maps and institutional affiliations.

## Supplementary Material

Supplementary Information

## Figures and Tables

**Figure 1:**
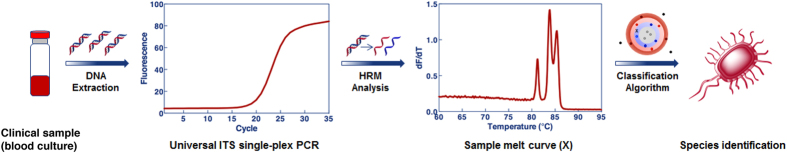
Workflow of our ITS HRM assay. The ITS HRM assay is performed on DNA extracted from clinical samples such as positive blood culture samples. Our novel classification algorithm allows generated melt curves to identify the bacterial pathogens by matching against a reference database. Same-colored dots represent replicates from the same species classified within their boundaries and black dots outside all boundaries represent replicates from species that is not in our reference database, whereas the unknown species in the test sample (X) is identified by the classification algorithm.

**Table 1:** Identification results of 59 positive clinical blood culture samples.

Positive Clinical Blood Culture Samples						
No.	Identification Results		ITS HRM Result			
	Culture Result	Sequencing Result	Top 1 Match		Top 2 Match	
			Classified	Misclassified	Classified	Misclassified
1	*Escherichia coli*	*Escherichia coli*	**✓**			
2	*Citrobacter freundii*	*Citrobacter freundii*	**✓**			
3	*Pseudomonas aeruginosa*	*Pseudomonas aeruginosa*	**✓**			
4	*Serratia marcescens*	*Serratia marcescens*	**✓**			
5	*Staphylococcus species*, coagulase negative	*Staphylococcus epidermis*	**✓**			
6	*Escherichia coli*	*Escherichia coli*	**✓**			
7	*Staphylococcus species*, coagulase negative	*Staphylococcus epidermis*	**✓**			
8	*Staphylococcus aureus*	*Staphylococcus aureus*	**✓**			
9	*Streptococcus group A*	*Streptococcus pyogenes*	**✓**			
10	*Streptococcus group C/G*	*Streptococcus dysgalactidae*	**✓**			
11	*Staphylococcus aureus*	*Staphylococcus aureus*	**✓**			
12	*Staphylococcus aureus*	*Staphylococcus aureus*	**✓**			
13	*Escherichia coli*	*Escherichia coli*	**✓**			
14	*Staphylococcus aureus*	*Staphylococcus aureus*	**✓**			
15	*Staphylococcus aureus*	*Staphylococcus aureus*	**✓**			
16	*Staphylococcus aureus*	*Staphylococcus aureus*	**✓**			
17	*Escherichia coli*	*Escherichia coli*	**✓**			
18	*Staphylococcus aureus*	*Staphylococcus aureus*	**✓**			
19	*Staphylococcus species*, coagulase negative	*Staphylococcus epidermis*	**✓**			
20	*Escherichia coli*	*Escherichia coli*	**✓**			
21	*Staphylococcus species*, coagulase negative	*Staphylococcus epidermis*	**✓**			
22	*Enterobacter cloacae*	*Enterobacter cloacae*	**✓**			
23	*Klebsiella oxytoca*	*Klebsiella oxytoca*	**✓**			
24	Streptococcus group B	*Streptococcus agalactiae*	**✓**			
25	*Escherichia coli*	*Escherichia coli*	**✓**			
26	*Escherichia coli*	*Escherichia coli*	**✓**			
27	*Acinetobacter baumannii/calcoaceticus complex*	*Acinetobacter baumannii*	**✓**			
28	*Staphylococcus species*, coagulase negative	*Staphylococcus epidermis*	**✓**			
29	*Staphylococcus aureus*	*Staphylococcus aureus*	**✓**			
30	*Staphylococcus aureus*	*Staphylococcus aureus*	**✓**			
31	*Staphylococcus species*, coagulase negative	*Staphylococcus hominis*	**✓**			
32	*Staphylococcus species*, coagulase negative	*Staphylococcus epidermis*	**✓**			
33	*Serratia marcescens*	*Serratia marcescens*	**✓**			
34	*Staphylococcus species*, coagulase negative	*Staphylococcus epidermis*	**✓**			
35	*Staphylococcus aureus*	*Staphylococcus aureus*	**✓**			
36	*Klebsiella pneumoniae*	*Klebsiella pneumoniae*	**✓**			
37	*Enterococcus faecalis*	*Enterococcus faecalis*	**✓**			
38	*Enterococcus faecalis*	*Enterococcus faecalis*	**✓**			
39	*Micrococcus luteus*	*Micrococcus luteus*	**✓**			
40	*Klebsiella pneumoniae ss. ozaenae*	*Klebsiella pneumoniae*	**✓**			
41	*Staphylococcus species*, coagulase negative	*Staphylococcus epidermis*	**✓**			
42	*Escherichia coli*	*Escherichia coli*	**✓**			
43	*Staphylococcus aureus*	*Staphylococcus aureus*	**✓**			
44	Viridans streptococcus group	*Streptococcus parasanguinis*	**✓**			
45	*Klebsiella pneumoniae*	*Klebsiella pneumoniae*	**✓**			
46	*Staphylococcus aureus*	*Staphylococcus aureus*	**✓**			
47	*Staphylococcus species*, coagulase negative	*Staphylococcus epidermis*	**✓**			
48	*Flavonifractor plautii*	*Flavonifractor plautii*	**✓**			
49	*Staphylococcus species*, coagulase negative	*Staphylococcus pettenkoferi*	U			
50	*Corynebacterium amycolatum*	*Corynebacterium sp.*	U			
51	*Fusobacterium necrophorum*	*Fusobacterium necrophorum*	U			
52	*Moraxella osloensis*	*Moraxella osloensis*	U			
53	*Bacteroides fragilis group*, not fragilis/thetaiotaomicron	*Bacteroides ovatus*	U			
54	*Klebsiella pneumoniae*	*Klebsiella pneumoniae*		*E. coli*	**✓**	
55	Viridans streptococcus group	*Streptococcus mitis*		*S. agalactiae*		*S. agalactiae*
56	*Staphylococcus species*, coagulase negative	*Staphylococcus hominis*		*S. epidermis*		*S. epidermis*
57	*Staphylococcus species*, coagulase negative	*Staphylococcus hominis*		*S. caprae*		*S. caprae*
58	*Serratia marcescens*	*Serratia marcescens*		U		U
59	*Klebsiella pneumoniae*	*Klebsiella pneumoniae*		U		U

U: Unknown (sample identified as an organism that was not in the database).
