# Supplementary material for: Microbial Typing by Machine Learned DNA Melt Signatures
Source: Sci Rep. 2017 Feb 6;7:42097. doi: 10.1038/srep42097 (PMC5292719; doi:10.1038/srep42097)
Supplement: Supplementary Information [file srep42097-s1.pdf]

Microbial Typing by Machine Learned DNA Melt Signatures

Nadya Andini[1], Bo Wang[2], Pornpat Athamanolap[3], Justin Hardick[4], Billie J. Masek[5], Simone Thair[1], Annie Hu[1], Gideon Avornu[5], Stephen Peterson[5], Steven Cogill[1], Richard E. Rothman[4,5], Karen C. Carroll[6], Charlotte A. Gaydos[4,5], Tza-Huei Wang[3,7], Serafim Batzoglou[2], Samuel Yang[1,*]

[1]Emergency Medicine, Stanford University, Stanford, California, 94305, USA.

[2]Computer Science, Stanford University, Stanford, California, 94305, USA.

[3]Biomedical Engineering, The Johns Hopkins University, Baltimore, Maryland, 21218, USA.

[4]Infectious Disease, Medicine, The Johns Hopkins University, Baltimore, Maryland, 21218, USA.

[5]Emergency Medicine, The Johns Hopkins University, Baltimore, Maryland, 21218, USA.

[6]Medical Microbiology, Pathology, The Johns Hopkins University, Baltimore, Maryland, 21218, USA.

[7]Mechanical Engineering, The Johns Hopkins University, Baltimore, Maryland, 21218, USA.

*corresponding.syang5@stanford.edu

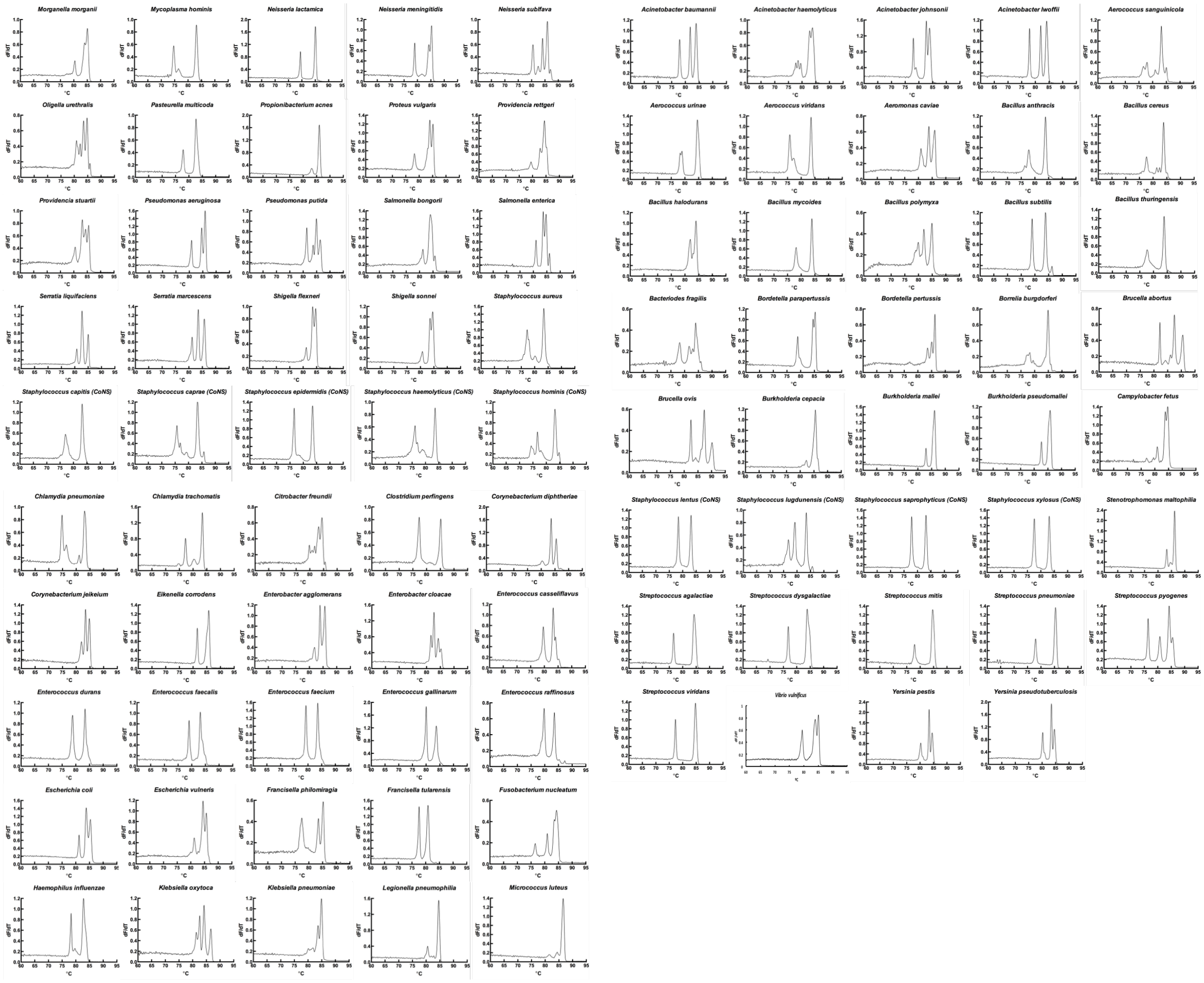

Supplementary Fig. S1. Individual derivative melt curves of 89 reference bacterial species.

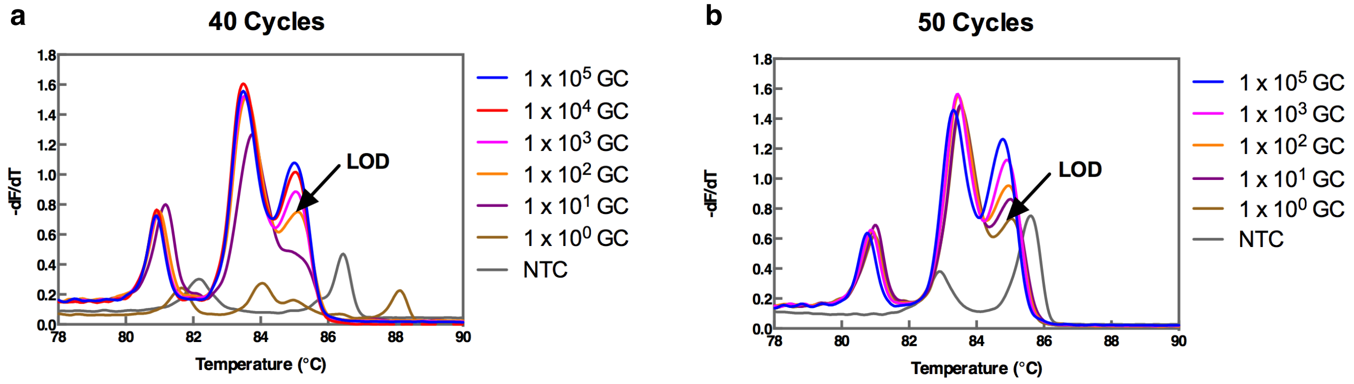

Supplementary Fig. S2. The limit of detection analysis of ITS PCR HRM. Serially diluted *E. coli* genomic DNA calculated based on its genome copies (GC) was amplified in a 40 (a) and a 50 (b)-cycle PCR targeting the ITS region. The PCR was immediately followed by HRM to produce corresponding derivative melt curves. The limit of detection (LOD) was determined to be the concentration where melt curve profile was maintained (arrows).

Supplementary Table S1. List of 89 reference bacterial species in the database.

Bacterial Species in Database			
Acinetobacter baumannii	*Burkholderia pseudomallei*	*Haemophilus influenzae*	*Shigella flexneri*
Acinetobacter haemolyticus	*Campylobacter fetus*	*Klebsiella oxytoca*	*Shigella sonnei*
Acinetobacter johnsonii	*Chlamydia pneumoniae*	*Klebsiella pneumoniae*	*Staphylococcus aureus*
Acinetobacter lwoffii	*Chlamydia trachomatis*	*Legionella pneumophila*	*Staphylococcus capitis (CoNS)*
Aerococcus sanguinicola	*Citrobacter freundii*	*Micrococcus luteus*	*Staphylococcus caprae (CoNS)*
Aerococcus urinae	*Clostridium perfingens*	*Morganella morganii*	*Staphylococcus epidermidis (CoNS)*
Aerococcus viridans	*Corynebacterium diphtheriae*	*Mycoplasma hominis*	*Staphylococcus haemolyticus (CoNS)*
Aeromonas caviae	*Corynebacterium jeikeium*	*Neisseria lactamica*	*Staphylococcus hominis (CoNS)*
Bacillus anthracis (2 strains)	*Eikenella corrodens*	*Neisseria meningitidis*	*Staphylococcus lentus (CoNS)*
Bacillus cereus	*Enterobacter agglomerans*	*Neisseria sublfava*	*Staphylococcus lugdunensis (CoNS)*
Bacillus halodurans	*Enterobacter cloacae*	*Oligella urethralis*	*Staphylococcus saprophyticus (CoNS)*
Bacillus mycoides	*Enterococcus casseliflavus*	*Pasteurella multicoda*	*Staphylococcus xylosus (CoNS)*
Bacillus polymyxa	*Enterococcus durans*	*Propionibacterium acnes*	*Stenotrophomonas maltophilia*
Bacillus subtilis	*Enterococcus faecalis*	*Proteus vulgaris*	*Streptococcus agalactiae*
Bacillus thuringensis	*Enterococcus faecium*	*Providencia rettgeri*	*Streptococcus dysgalactiae*
Bacteriodes fragilis	*Enterococcus gallinarum*	*Providencia stuartii*	*Streptococcus parasanguinis*
Bordetella parapertussis	*Enterococcus raffinosus*	*Pseudomonas aeruginosa*	*Streptococcus pneumoniae* (5 strains)
Bordetella pertussis	*Escherichia coli*	*Pseudomonas putida*	*Streptococcus pyogenes*
Borrelia burgdorferi	*Escherichia vulneris*	*Salmonella bongorii*	*Streptococcus anginosus*
Brucella abortus	*Francisella philomiragia*	*Salmonella enterica Enteritidis*	*Vibrio fluvialis*
Brucella ovis	*Francisella tularensis*	*Serratia liquifaciens*	*Yersinia pestis* (2 strains)
Burkholderia cepacia	*Fusobacterium nucleatum*	*Serratia marcescens*	*Yersinia pseudotuberculosis*
Burkholderia mallei			

SUPPLEMENTARY METHODS

1. Naïve Bayes

In this section, we present details about the proposed adaptive Naïve Bayes algorithm. Given C species in the reference dataset, and for the i-th species C_i, we have N_i number of training samples. For any new unknown test sample x, we aim to calculate the posteriori probability via Bayes' theorem:

$$p(C_k|x) = \frac{p(C_k)p(x|C_k)}{p(x)}$$

where $p(C_k)$ is the prior for the k-th species, and $p(x|C_k)$ is the likelihood function given all the training samples in the k-th species.

The prior information is assumed to be homogeneous:

$$p(C_k) = \frac{1}{C}$$

The likelihood function is calculated with a Gaussian distribution:

$$p(x|C_k) = \sum_{x'_j \epsilon C_k} \frac{1}{\sqrt{2\pi\alpha}} \exp(-\frac{D\left(x, x'_j\right)}{2\alpha}).$$

The essence in the algorithm lies in the way we calculate the distance $D(x, x'_j)$. This measures the similarity between curve shapes for the test sample and training reference.

Assume for a test species, denoted as $S_t = \{S_t^1, S_t^2, \ldots, S_t^m\}$ where m is the number of replicates in this species. We want to achieve a consensus prediction of whether this species falls into any species category from the reference panel. We assume each replicate of same importance, so we just average the final posteriori probability of each replicate to obtain the prediction for the test species:

$$p(C_k|S_t) = \frac{1}{m} \sum_{j}^{m} p(C_k|S_t^j)$$

2. Curve Similarity Calculation

There are three steps in the calculation of curve similarity. First, we align each curve according to the temperature of 53°C. This guarantees each curve is well-aligned and thus high accuracy in the following

curve similarity calculation. Second, we apply Hilbert Transformation on the curves. Hilbert transformation is a convolution process on the curve:

$$H(f)(t) = \frac{1}{\pi}\int_{-\bowtie}^{\bowtie} \frac{f(\tau)}{t-\tau} d\tau$$

where f(t) denotes the curve we have. The output of Hilbert transformation is a complex function where the real part is the original input and the complex part denotes the transformed domain. We calculate the distance between two curves by combining the two parts as follows:

$$D(f,g) = \sum_t ||\boldsymbol{real}(H(f)(t)) - \boldsymbol{real}(H(g)(t))||^2 + \sum_t ||\boldsymbol{complex}(H(f)(t)) - \boldsymbol{complex}(H(g)(t))||^2$$

where f and g represent two curves.

3. Details in predicting out-of-reference samples

To distinguish whether the test target belongs to any species in the reference panel, we adapt the original Naïve Bayes to accommodate the prediction of out-of-reference samples. Assume for a test species, denoted as $S_t = \{S_t^1, S_t^2, \dots, S_t^m\}$ where m is the number of replicates in this species. First, for each replicate, we assign a prior probability to be out-of-reference sample by looking at the curve region between temperature 52.5°C to 53.5°C. This would give us some knowledge about whether this replicate is an outlier because most of outlier curves will generate some unusual peak curves in this temperature region. Further, when we apply Naïve Bayes, we assign the posteriori probability to be out-of-reference by adding a gated function that if the following quantile is below some threshold:

$$P(S_t \in C_0) = I\{max_k p(S_t|C_k) < \theta\}.$$

we set θ = 0.3 in our experiments.
